# Rapid phase contrast MRI with minimum time gradient waveform design using convex optimization

**DOI:** 10.1186/1532-429X-16-S1-W7

**Published:** 2014-01-16

**Authors:** Matthew J Middione, Holden H Wu, Daniel B Ennis

**Affiliations:** 1 Department of Radiological Sciences, University of California, Los Angeles, Los Angeles, California, USA; 2 Biomedical Physics Interdepartmental Program, University of California, Los Angeles, Los Angeles, California, USA

## Background

Phase contrast MRI (PC-MRI) sequences conventionally use triangular and trapezoidal gradient waveforms to construct flow compensated and flow encoded (FCFE) [1] or Bipolar [2] velocity encoding gradients. This approach does not make optimal use of the available gradient hardware for all time points, but convex optimization (CVX) can be used to design minimum-duration, arbitrarily shaped gradient waveforms subject to gradient hardware and pulse sequence constraints (e.g. target gradient moments and imaging parameters) [3]. Our objective was to quantitatively evaluate CVX PC-MRI measurement accuracy and sequence efficiency.

## Methods

**Figure 1:**
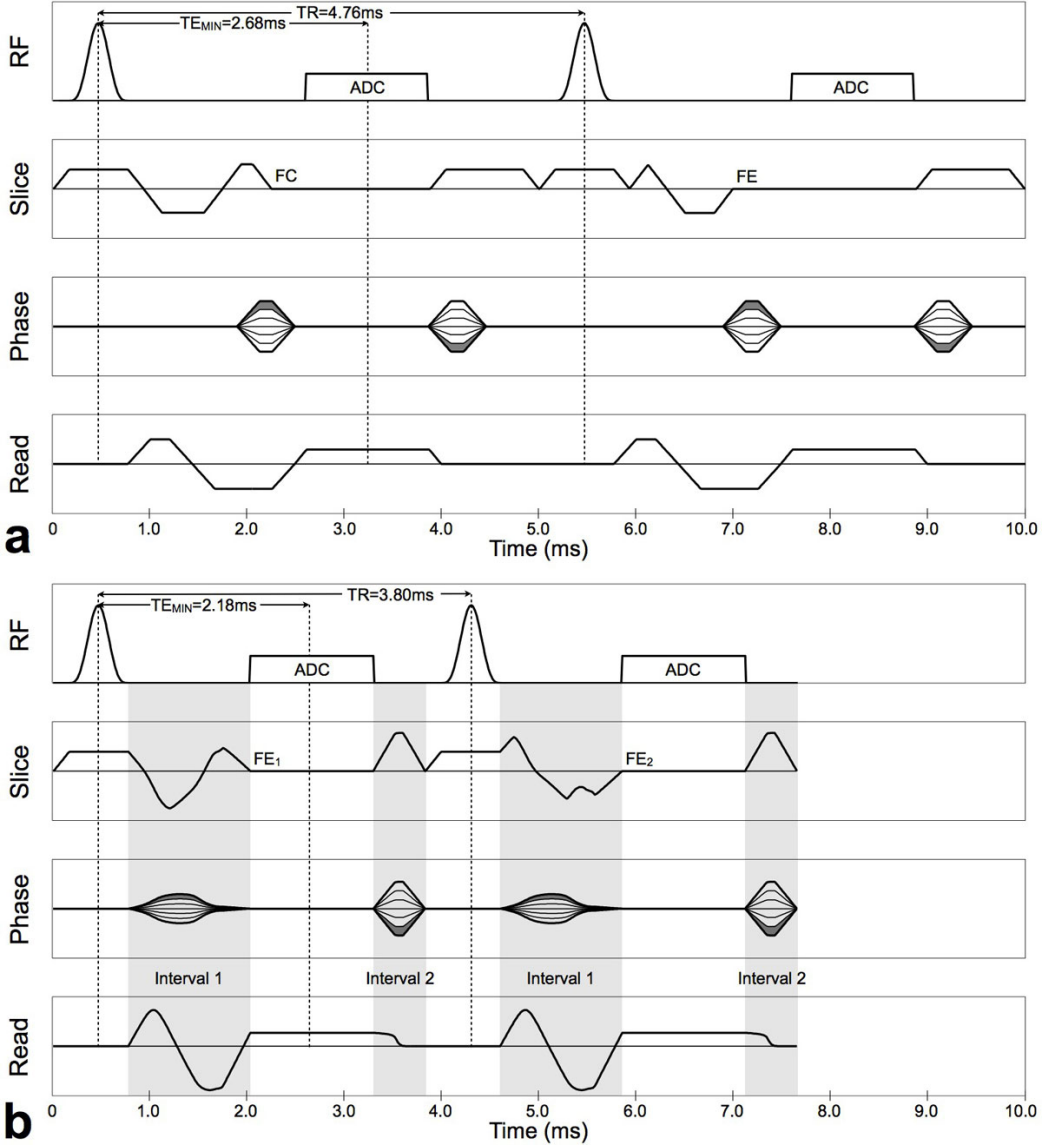
**Pulse sequence diagrams for 2D through-plane PC-MRI sequences for VENC = 150 cm/s**: (a) conventional flow compensated and flow encoded (FCFE) velocity encoded sequence (TE_MIN _= 2.68 ms, 1.8 mm × 1.8 mm spatial resolution, 38 ms temporal resolution) and (b) convex gradient optimized (CVX) velocity encoded sequence (TE_MIN _= 2.18 ms, 1.8 mm × 1.8 mm spatial resolution, 30 ms temporal resolution). Two separate CVX optimizations are conducted for each sequence as indicated by the gray regions within the pulse sequence diagrams. The two slice select gradients, the flow compensated readout gradient, and the phase encoding gradient are optimized during the time between RF transmission and data acquisition (Interval 1). The spoiler, readout ramp down, and phase encode rewinder gradients are optimized during the time between the end of data acquisition and the end of the TR (Interval 2).

CVX was used for all gradient waveforms during Interval-1 (end of the slice-select gradient plateau to the beginning of the readout gradient plateau) and Interval-2 (end of the readout gradient plateau to the end of the TR, Figure 1), subject to (G_x_^2^+G_y_^2^+G_z_^2^)^0.5^≤38 mT/m and (SR_x_^2^+SR_y_^2^+SR_z_^2^)^0.5^≤170 mT/m/ms. CVX PC-MRI also used asymmetric velocity encoding [4] for 2D through-plane velocity encoding to further improve sequence efficiency. Based on our recent evaluation of gradient spoiling in PC-MRI [6], the CVX sequence used 4π gradient spoiling while the FCFE sequence used a vendor hard-coded 9π gradient spoiling. A computer controlled flow phantom (sine wave with peak velocity of 150 cm/s) was used to validate that the arbitrarily shaped gradients used in CVX did not lead to measurement errors compared to FCFE. Bland-Altman analysis (reported as bias [lower 95%-CI, upper 95%-CI]) was used to compare FCFE and CVX velocity measurements with identical scan parameters (TE = 2.68 ms for 1.8 mm × 1.8 mm and 38 ms spatiotemporal resolution). Over a wide range of VENCs the minimum achievable TE_MIN _and TR_MIN _were compared for FCFE, Bipolar, and CVX using an otherwise fixed protocol. Sequence efficiency (E), defined as the ratio of the readout duration to the TR [5], was calculated for each VENC and sequence. Relative sequence efficiencies were compared as [E_CVX_-E_FCFE_]/E_CVX _and [E_CVX_-E_Bipolar_]/E_CVX_.

## Results

**Figure 2:**
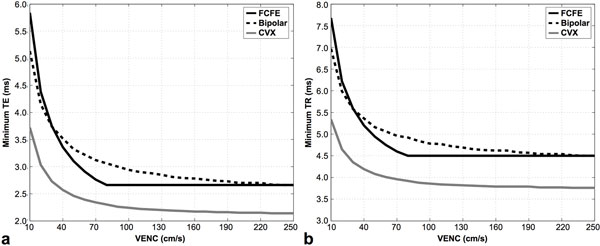
**Minimum achievable TE (a) and TR (b) plotted as a function of the target VENC for conventional flow compensated and flow encoded velocity encoding (FCFE; solid black), Bipolar velocity encoding (black dashed), and convex gradient optimized PC-MRI (CVX; solid gray)**. As the VENC increases, a theoretical minimum TE/TR is reached for FCFE and Bipolar because of limitations associated with the plateau of the readout gradient overlapping with the velocity encoding gradients gradient. The CVX sequence constructs asymmetrically weighted velocity encoding gradients and a flow compensated readout gradient to be of equal duration, thus overcoming this limitation and offering shorter values for TE/TR compared FCFE and Bipolar. All other imaging parameters were held constant (1.8 mm × 1.8 mm × 5 mm resolution, 30° flip angle, 814 Hz/px receiver bandwidth, 600 μs RF pulse width, 4π gradient spoiling for CVX and a vendor hard-coded 9π gradient spoiling for FCFE and Bipolar).

The Bland-Altman bias was 0.28 cm/s [-7.14 cm/s, 7.69 cm/s]. CVX produces the shortest TE_MIN _and TR_MIN _for every VENC (Figure 2). This leads to an increase in relative sequence efficiency by an average of 20 ± 6% (15% [min], 44% [max]) compared to FCFE and 24 ± 3% (20%, 31%) compared to Bipolar.

## Conclusions

The Bland-Altman results indicate excellent agreement between CVX and FCFE (very low measurement bias and narrow 95%-CIs). Calculations show that E_CVX _can be increased by as much as 44%. Improvements in E_CVX _can be used to improve spatiotemporal resolution for a fixed breath hold duration or to reduce breath hold duration for an otherwise fixed protocol.

## Funding

This work was enabled by research support from Siemens Medical Solutions and the Department of Radiological Sciences to DBE.
